# Relative power and coherence of EEG series are related to amnestic mild cognitive impairment in diabetes

**DOI:** 10.3389/fnagi.2014.00011

**Published:** 2014-02-04

**Authors:** Zhijie Bian, Qiuli Li, Lei Wang, Chengbiao Lu, Shimin Yin, Xiaoli Li

**Affiliations:** ^1^Institute of Electrical Engineering, Yanshan UniversityQinhuangdao, China; ^2^Department of Neurology, The Second Artillery General Hospital of PLABeijing, China; ^3^State Key Laboratory of Cognitive Neuroscience and Learning and IDG/McGovern Institute for Brain Research, Beijing Normal UniversityBeijing, China; ^4^Center for Collaboration and Innovation in Brain and Learning Sciences, Beijing Normal UniversityBeijing, China

**Keywords:** resting-state EEG, amnestic mild cognitive impairment, diabetes, relative power, coherence

## Abstract

**Objective:** Diabetes is a risk factor for dementia and mild cognitive impairment. The aim of this study was to investigate whether some features of resting-state EEG (rsEEG) could be applied as a biomarker to distinguish the subjects with amnestic mild cognitive impairment (aMCI) from normal cognitive function in type 2 diabetes.

**Materials and Methods:** In this study, 28 patients with type 2 diabetes (16 aMCI patients and 12 controls) were investigated. Recording of the rsEEG series and neuropsychological assessments were performed. The rsEEG signal was first decomposed into delta, theta, alpha, beta, gamma frequency bands. The relative power of each given band/sum of power and the coherence of waves from different brain areas were calculated. The extracted features from rsEEG and neuropsychological assessments were analyzed as well.

**Results:** The main findings of this study were that: (1) compared with the control group, the ratios of power in theta band [P(theta)] vs. power in alpha band [P(alpha)] [P(theta)/P(alpha)] in the frontal region and left temporal region were significantly higher for aMCI, and (2) for aMCI, the alpha coherences in posterior, fronto-right temporal, fronto-posterior, right temporo-posterior were decreased; the theta coherences in left central-right central (LC-RC) and left posterior-right posterior (LP-RP) regions were also decreased; but the delta coherences in left temporal-right temporal (LT-RT) region were increased.

**Conclusion:** The proposed indexes from rsEEG recordings could be employed to track cognitive function of diabetic patients and also to help in the diagnosis of those who develop aMCI.

## Introduction

That diabetes affects cognitive function was first reported by Mile and Root in the 1920s (Miles and Root, 1922). Diabetes patients were found to have neuronal death and axonal degeneration, a concept of “diabetic encephalopathy” was thus raised in Gispen and Biessels (2000). Epidemiological data showed that the diabetic patients was associated with a 1.5–2.5-fold increased risk of dementia (Strachan et al., 2011).

The MCI is defined as impairment in cognitive functions, particularly memory, with otherwise normal performance of activities of daily living. The MCI lies between and overlaps normal aging and Alzheimer's disease (AD) and is now recognized to be a risk factor for AD (Levey et al., 2006) or an early manifestation of the disease (Morris, 2005). The MCI includes two subtypes: amnestic mild cognitive impairment (aMCI) and non-amnestic MCI (na-MCI). The aMCI patients are the high-risk groups of AD. The percent change of conversion from aMCI to AD was 54% and the conversion duration from initial diagnosis of aMCI to dementia was 28 ± 12 months (Seo et al., 2012).

Type 2 diabetes, is characterized by high blood glucose in the context of insulin resistance and relative insulin deficiency (Kumar et al., 2005). Cognitive impairment such as learning and memory deficiency was seen in type 2 diabetes (Peila et al., 2002). The diabetes may be associated with increased risk of both aMCI and na-MCI (Shimada et al., 2010; Roberts et al., 2014). Therefore, it is critical to explore methods to detect the aMCI of diabetes patients, so that the early interventions to these patients can be provided.

Petersen described the MCI based on clinical criteria (Petersen, 2004), however current proposals also include biomarkers (Albert et al., 2011). By using the ligand-based positron emission tomography (PET), abnormal dosages of the beta amyloid to tau ratio in cerebrospinal fluid (CSF) and deposition of beta amyloid in the brain can be used to diagnose the prodromal stages of AD in MCI subjects; moreover, the neurodegeneration such as atrophy of the hippocampus on magnetic resonance imaging (MRI) and hypometabolism of the posterior cingulate/precuneus, parietal and temporal regions revealed by fluorodeoxyglucose (FDG)-PET are all useful biomarkers for diagnosis of prodromal stages of AD (Albert et al., 2011). However, the sensitivity and specificity of these biomarkers were different for the different international databases (Toussaint et al., 2012; Takahashi et al., 2013). Moreover, the CSF markers are invasive, the PET markers are costly and expose patients to radiation, and the MRI markers of hippocampus volume are relatively expensive for serial screening of large elderly populations at risk for AD; therefore, a non-invasive and cost-effective tool is needed. It was demonstrated that the cerebral EEG rhythms can reflect the underlying brain network activity (Steriade, 2006), and the resting-state EEG (rsEEG) can be used to perform serial examinations for neurological evolution (Rossini et al., 2007; Schmidt et al., 2013). Recent studies have investigated the rsEEG rhythms in MCI and AD subjects, which may be a promising approach to assess MCI subjects (Babiloni et al., 2014). This technique is low-cost, easy to use, presents a high temporal resolution and is non-invasive.

Spectral power of EEG series and their correlations with neuropsychological tests can provide valuable information in distinguishing normal and diseased brain function (Roh et al., 2011). The theta and delta spectral power tended to increase in the selected brain regions according to cognitive impairment from normal through aMCI to AD, whereas alpha and beta2 power showed a decreasing tendency (Roh et al., 2011). In particular, relative power has been used as a feature to classify the MCI and mild AD patients from age-matched controls (Jelic et al., 1996; Dauwels et al., 2011). In addition, the EEG coherence has been used to evaluate the functionality of cortical connections and to provide information about the synchronization of the regional cortical activity in AD. In Sankari et al. (2011), it was found that decreased coherence indicates a decline in cortical connectivity in AD, which suggests that the coherence of EEG signals have potentials in differentiation of healthy elderly from AD patients. Power and coherence were considered as features for the classification of the AD and control groups, since the classification accuracy reached to 89% (Strijers et al., 1997; Stevens et al., 2001). Therefore, rsEEG indexes may have the potential as a biomarker to distinct the aMCI or AD from controls. In order to seek a better early diagnosis method of aMCI for patients with diabetes, rsEEG indexes (relative power and coherence in different regions and frequencies) were investigated in this study.

## Materials and methods

### Participants

In this study the participants were 28 right-handed type 2 diabetes patients who satisfied the diagnosis criteria for diabetes (American Diabetes Association, 2013), and they were all voluntary and more than 50 years old. These participants were divided into 2 groups: aMCIs and controls. The aMCI group consisted of 16 patients (5 males and 11 females; mean age 69.7 ± 8.4 years, range from 52 to 84 years; mean years of diabetes 9.3 ± 2.4 years, range from 1 to 20 years; mean years of education 12.9 ± 1.8 years, range from 6 to 16 years), and the control group consisted of 12 patients (6 males and 6 females; mean age 73.3 ± 4.6 years, range from 63 to 80 years; mean years of diabetes 14.0 ± 3.1 years, range from 1 to 30 years; mean years of education 13.8 ± 3.0 years, range from 9 to 19 years). Both groups were matched in age, diabetes duration and education level, but not in gender.

The study was approved by the local ethics committee and all patients gave written informed consent. The experiment was conducted in accordance with the Declaration of Helsinki (1964) and was approved by the Beijing Normal University ethics committee.

### Neuropsychological tests and inclusion criteria

Based on traditional MMSE (Folstein et al., 1975), and considering the China National State, a modified MMSE proposed by Shanghai Mental Health Center in China (Jia, 2010) was performed to all diabetic participants in this study. The cut-off score for absence of dementia was 24 points for high school and above, 20 points for the primary, and 17 for the illiteracy participants, so the scores of the two groups were all more than 24 points, which included MCI and normal function participants. The MoCA uses a cut-off score 26 points for MCI (Nasreddine et al., 2005). Compared with MMSE, the MoCA appears to be a better screening tool for MCI in the diabetic population because it possesses higher sensitivity (67%) (Alagiakrishnan et al., 2013). Therefore, in this study cognitive disorders were further screened by using MoCA after the preliminary screening of MMSE, which was only used to rule out AD preliminarily.

In this study, besides MMSE and MoCA, Auditory Verbal Learning Test (AVLT) (AVLT-Immediate recall, AVLT-Delayed recall, AVLT-Delayed recognition) (Carlesimo et al., 1996), Wechsler Adult Intelligence Scale Digit Span Test (WAIS-DST) (Orsini et al., 1987), Boston Naming Test (BNT), Trail Making Test (Reitan, 1958), Verbal Fluency Test (Novelli, 1986), Daily Living Test (Lawton and Brody, 1969) were performed to each subject.

The participants were all type 2 diabetes patients, whose vision and hearing were able to complete clinical trials. They underwent MRI examination to rule out the organic brain disease. The depression that can cause cognitive impairment was ruled out using DSM IV criteria for depression (American Psychiatric Association, 1994). No patients in either group reported a history of mental illness, systemic disease (such as liver and kidney dysfunction, heart disease and thyroid disease) and nervous system disease (such as cerebrovascular disease, traumatic brain injury, epilepsy, encephalitis, hydrocephalus, brain tumors, multiple sclerosis, radiation injury) that resulting to cognitive impairment.

The diabetic aMCI patients satisfied the criteria (Petersen, 2004) for the study diagnosis of aMCI. The inclusion criteria were as follows: (1) memory complaint usually coming from the patients or their family; (2) objective memory impairment for age defined by performances ≥1.5 standard deviation below the mean value of age- and education-matched controls for the Auditory Verbal Learning Test (Carlesimo et al., 1996); (3) essentially preserved general cognitive function tested using MMSE and MoCA; (4) normal activities of daily living evidenced by Activity of Daily Living Scale (Lawton and Brody, 1969); (5) not demented [the dementia was ruled out by DSM IV criteria for dementia (American Psychiatric Association, 1994)].

### EEG recording

The experiment was performed in the Department of Neurology, General Hospital of Second Artillery Corps of PLA, Beijing, China. The participants were asked to wash and brush their hair before the application of the Geodesic Sensor Net (GSN) to their head. During recordings, they were asked to close their eyes and sit in a comfortable armchair, keeping relaxed and awake for 5 min in a quiet-dim room, with room temperature keeping at 23 ± 2°C.

The EEG data recording was performed with a high-density 128-channel EGI system of Net Amps 300 amplifiers (Electrical Geodesics Inc. [EGI], Eugene, OR). The EEG was recorded continuously with a 128-channel GSN using the vertex sensor (Cz) as the reference electrode. Direct current acquisition was used and the data were sampled at 1000 Hz during recording. The impedances of all electrodes were kept below 50 kΩ, as recommended for this type of amplifiers by EGI guidelines.

### EEG preprocessing

The recorded EEG data were analyzed off-line using NetStation 4.5 software (Electrical Geodesics). First, a band-pass filter of 1–45 Hz was applied; then the data were re-referenced to the average of 57 (left mastoid process) and 100 (right mastoid process) sensors, and the data were re-sampled to 500 Hz. The artifacts (such as ocular and muscular) were removed by visual inspection of the raw EEG data. Finally EEG recordings of 3 min were segmented for further analysis.

**Figure 1:**
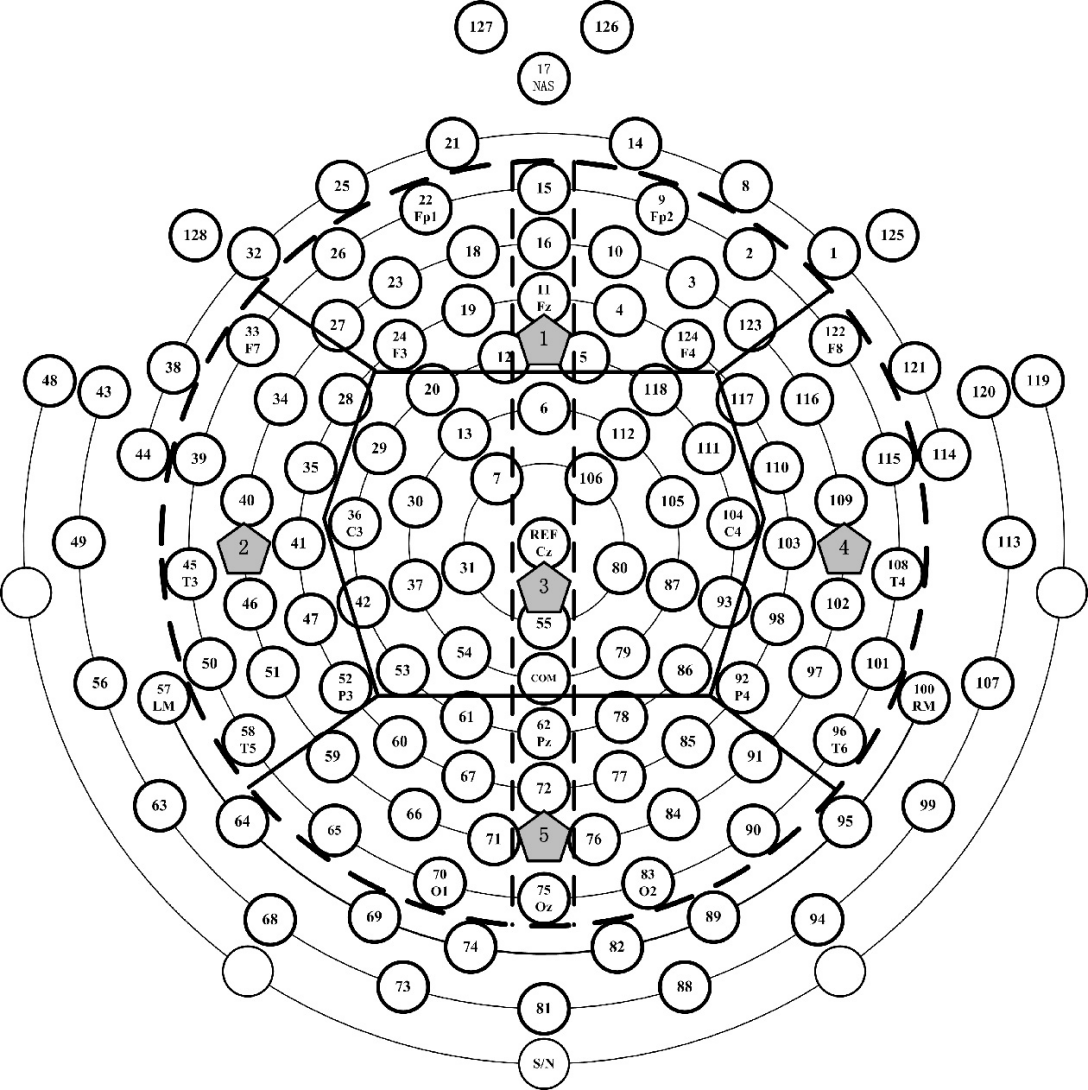
**The electrodes distribution of 128-channel Geodesic Sensor Net interested electrodes' partition**. The interested electrodes were those inside the black dotted line. Thick solid lines divided the interested electrodes into 5 regions: the number 1, 2, 3, 4, and 5 denote the frontal, left temporal, central, right temporal and posterior regions, respectively. Vertical dotted line divided the brain into left and right hemispheres (LH and RH), left frontal (LF) and right frontal (RF), left central (LC) and right central (RC), and left posterior (LP) and right posterior (RP).

The data was recorded using the 128-channel GSN, but in this study, the interested electrodes were circled inside the dashed line (see Figure 1), which can throughout the whole brain area. In order to detect EEG power in different regions and inter-/intra-regions coherence, the brain were divided into five regions: frontal (F), left temporal (LT), central (C), right temporal (RT), and posterior (P). For the aim to estimate the left and right hemispheres paired-electrodes coherence, the vertical dashed line (see Figure 1) divided the brain into left and right hemispheres (LH and RH) and the frontal region was divided into left frontal (LF) and right frontal (RF), the central region into left central (LC) and right central (RC), the posterior into left posterior (LP) and right posterior (RP).

### EEG data analysis

In this study, power and coherence were calculated at the five frequency bands: delta (1–4 Hz), theta (4–8 Hz), alpha (8–13 Hz), beta (13–30 Hz), gamma (30–45 Hz). For each band, the relative power and the coherence were obtained. The EEG data of 10 s were divided into overlapping segments using periodic 2-s hamming windows with 50% overlap, then the power spectral density (PSD) and the coherence of were computed using pwelch method (Welch, 1967). Outliers rejection was performed by means of a generalized extreme studentized deviate (GESD) (Seem, 2007) for all epochs.

#### Relative power

The relative power of each given band/sum of power from 1 to 45 Hz was calculated by

$$RP\text{​}\left(f_{1},f_{2}\right)=\frac{P\text{​}\left(f_{1},f_{2}\right)}{P(1,45)}\times 100\%$$

where *P*(·) indicates the power, *RP*(·) indicates the relative power, and *f*_1_, *f*_2_ indicate the low and high frequency, respectively.

The ratios of power for different frequency bands in each electrode was computed for possible pairs of frequency bands, such as P(delta)/P(theta) [or /P(alpha), or /P(beta), or /P(gamma)], P(theta)/P(alpha) [or /P(beta), or /P(gamma)], P(alpha)/P(beta) [or /P(gamma)] and P(beta)/P(gamma).

The relative power for each band and the ratios of power for different frequency bands were averaged in each region.

#### Coherence

In this study, the magnitude squared coherence *C*_*xy*_ of signals *x* and *y* was estimated by using the PSD (*P*_*xx*_ and *P*_*yy*_) and the cross PSD (*P*_*xy*_), it is

$$C_{xy}(f)=\frac{{\left|P_{xy}(f)\right|}^{2}}{P_{xx}(f)*P_{yy}(f)}$$

where *f* is the frequency. In this study, Welch's averaged (a modified period-gram method) (Welch, 1967) was used to compute the coherence. Compared with other power spectra estimating methods, it is better to against noise. In the calculation, the frequency resolution was 0.1, and a coherence matrix {*C_xy_*} for the whole frequency band (1–45 Hz) was obtained. Then the average coherence values for each pair of electrodes over each EEG band were computed by

$$A_{xy}=\frac{1}{U-L}\int_{L}^{U}C_{xy}(f)df$$

where *U* and *L* were the upper and the lower bound frequencies for each band. The coherence of each pair of electrodes over the five frequency bands for each subject was calculated. After outlier rejection, the remained epochs were averaged. From this point, we called the averaged coherence as coherence.

The intra-/inter-coherence in the five different regions and the paired-electrodes coherence (e.g., 22-9, 26-2, 45-108 and so on) over the left and right hemispheres were calculated.

### Statistical analysis

In this study, Wilcoxon rank sum test was conducted at the 5% significance level including the EEG relative power of each brain area, the inter hemispheric coherence along with the intra-/inter-coherence in the five regions and the neuropsychological scores between aMCI and controls.

In order to determine whether rsEEG can be biomarkers to detect aMCI in diabetes, the correlations between the significantly different neuropsychological items and the EEG indexes over significantly different regions in significantly different bands were analyzed in the diabetic aMCI and control groups. Pearson's linear correlation was employed in this study.

## Results

### Neuropsychological tests

The neuropsychological assessment scores of the aMCIs and controls (the *p* values for the tested items) were shown in Table 1. There were significant differences in scores of MoCA, AVLT-Immediate recall, AVLT-Delayed recall, AVLT-Delayed recognition, BNT, WAIS-DST. The scores of MMSE, Trail Making Test, Verbal Fluency Test, and Daily Living Skills Test were lower in aMCI patients than in controls, but these differences were not statistically significant.

**Table 1:** **Neuropsychological assessment scores (mean ± s.e.m.) and *p* values for the tested items in the diabetic aMCI and control groups**.

**Items**	**aMCI (*n* = 16)**	**Control (*n* = 12)**	***P* values**
MMSE	27.9 ± 0.5	28.8 ± 0.2	0.529
MoCA	22.4 ± 0.5	27.0 ± 0.3	*p* < 0.0001^***^
AVLT-Immediate recall	5.4 ± 0.4	7.6 ± 0.5	0.002^*^
AVLT- Delayed recall	4.3 ± 0.9	8.8 ± 1.0	0.005^**^
AVLT- Delayed recognition	10.9 ± 0.9	13.8 ± 0.3	0.008^**^
BNT	18.7 ± 0.4	19.8 ± 0.1	0.048^*^
WAIS-DST	11.5 ± 0.7	14.6 ± 0.6	0.002^**^
Trail Making Test1	65.4 ± 5.3	58.8 ± 4.9	0.354
Trail Making Test2	112.3 ± 14.3	99.9 ± 10.0	0.699
Verbal Fluency Test	15.6 ± 0.8	18.0 ± 0.1	0.099
Activity of Daily Living Scale	14	14	—

*Key: MMSE, Mini-Mental State Examination; MoCA, Montreal Cognitive Assessment; AVLT, Auditory Verbal Learning Test; BNT, Boston Naming Test; WAIS-DST, Wechsler Adult Intelligence Scale Digit Span Test; aMCI, amnestic mild cognitive impairment; MCI, mild cognitive impairment; s.e.m., Standard Error of the Mean*.

* p < 0.05;

** p < 0.01;

*** *p < 0.001*.

### Relative power

**Figure 2:**
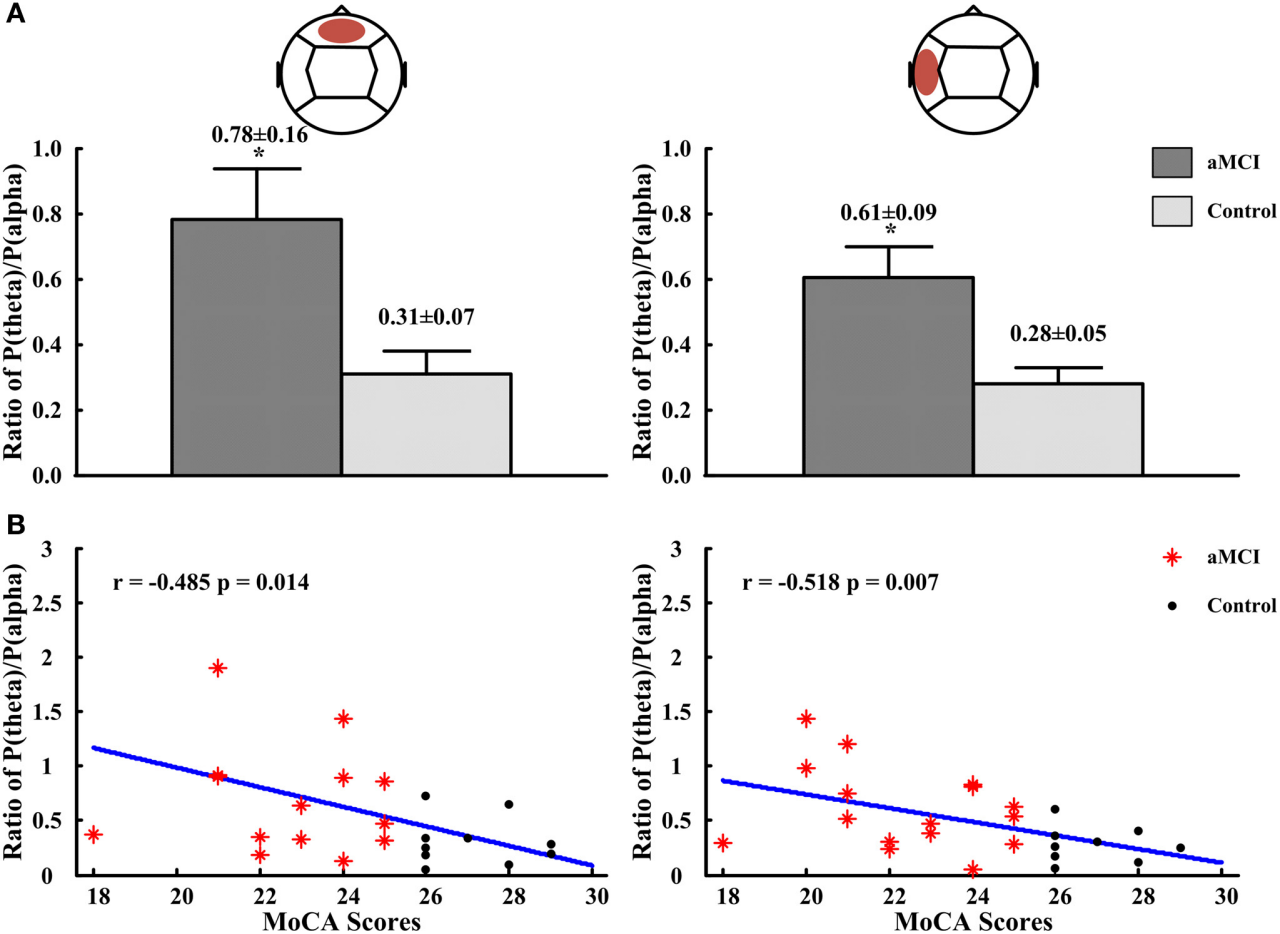
**The ratios of P(theta)/P(alpha) in frontal and left temporal regions and the correlations between the ratios of P(theta)/P(alpha) and scores of MMSE and MoCA. (A)** The ratios of P(theta)/P(alpha) in frontal region (left panel) and in left temporal region (right panel). **(B)** The correlation between ratios of P(theta)/P(alpha) and scores of MMSE in frontal region (left panel) and in left temporal region (right panel). ^*^p < 0.05.

The relative power for all frequency bands were not significantly different in diabetes between the aMCI and control groups, but the ratios of P(theta)/P(alpha) in the frontal and temporal regions showed statistically significant differences. Compared to the control group, the ratios of P(theta)/P(alpha) in the frontal region (aMCI: 0.78 ± 0.16, control: 0.31 ± 0.07; *p* < 0.05) and the left temporal region (aMCI: 0.61 ± 0.09, control: 0.28 ± 0.05; *p* < 0.05) were significantly higher in the subjects with aMCI (see Figure 2A). For the ratios obtained in other regions and at other frequency bands there are no differences between both groups, the significant ratios of relative power obtained were then correlated with those neuropsychological measures in which there were differences between controls and aMCI subjects. Figure 2B showed that the ratios of P(theta)/P(alpha) were negatively correlated to the scores of MoCA in the frontal (*r* = −0.485, *p* = 0.014) and left temporal (*r* = −0.518, *p* = 0.007) regions. There were no significant correlations between ratios of relative power and neuropsychological tests.

### Coherence

**Figure 3:**
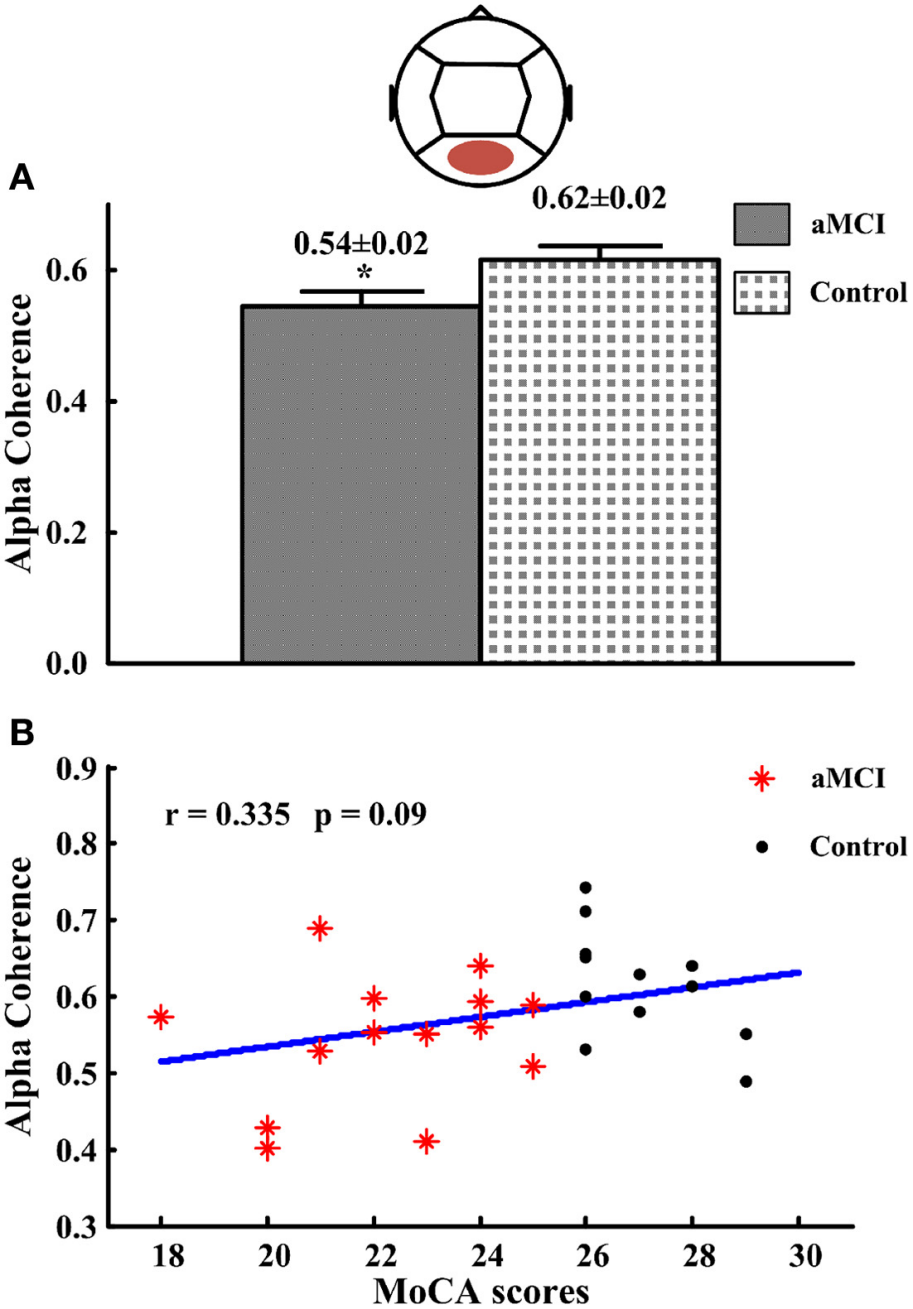
**The alpha coherence in posterior region and the correlations between the alpha coherence and the scores of MoCA. (A)** Alpha coherence in posterior region. **(B)** The correlations between the alpha coherences and the scores of MoCA. ^*^p < 0.05.

In posterior region (intra-region), alpha coherence was lower for subjects with aMCI (0.54 ± 0.02; *p* < 0.05) compared to the controls (0.62 ± 0.02) (see Figure 3A). There were no significant differences for coherence at other frequency bands or regions. Then the correlations between significant different intra-region coherence and neuropsychological tests were analyzed. No significant correlation between the alpha coherence and neuropsychological tests was found, including the scores of MoCA (*r* = 0.335, *p* = 0.09) (see Figure 3B).

**Figure 4:**
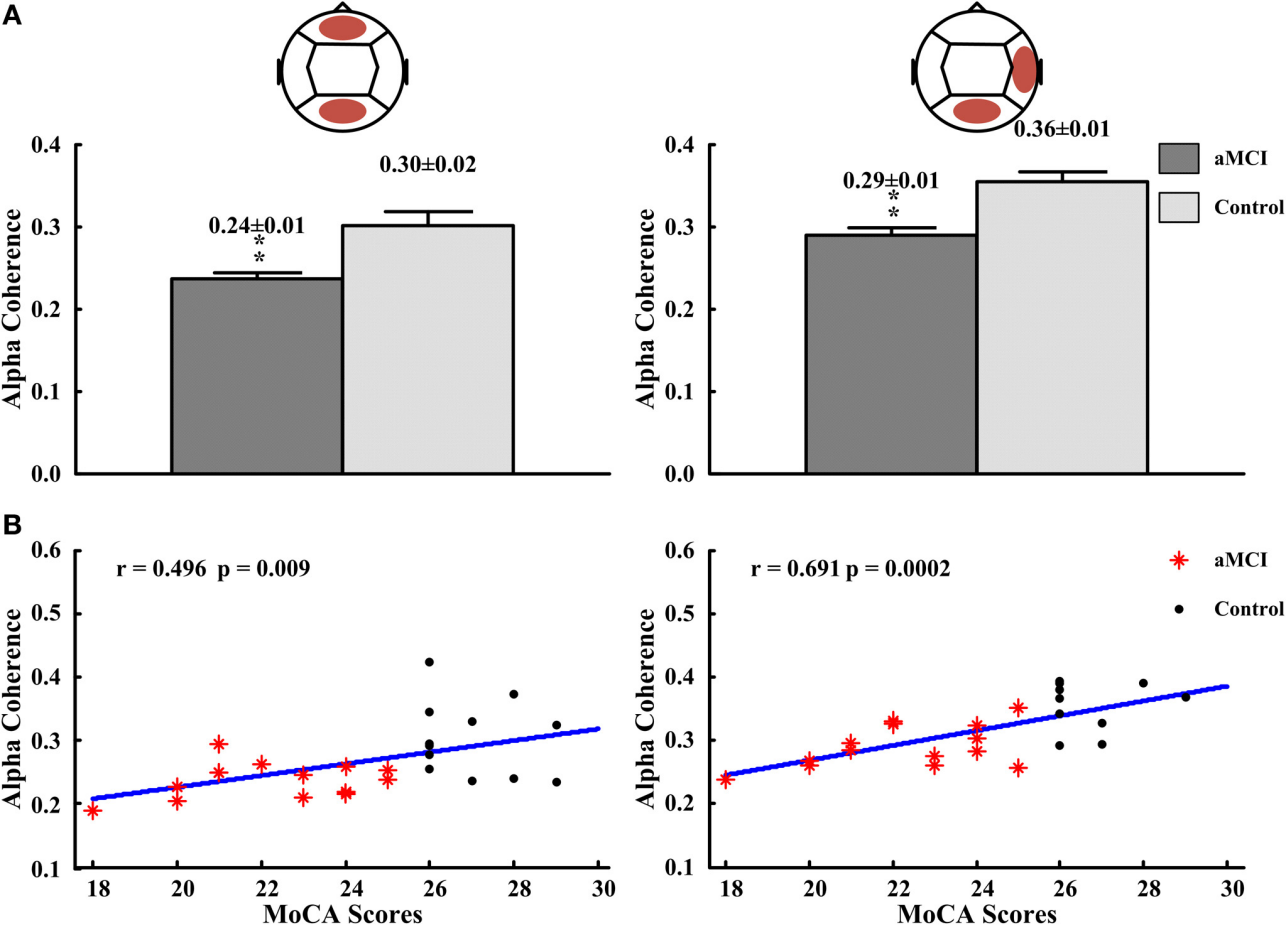
**The alpha coherences in fronto-posterior and right temporal-posterior regions and the correlations between the alpha coherences and the scores of MoCA. (A)** The alpha coherences in fronto-posterior (left panel) and right temporal-posterior (right panel) regions. **(B)** The correlation between alpha coherences and scores of MoCA in fronto-posterior (left panel) and right temporal-posterior (right panel) regions. ^**^p < 0.01.

In inter-regions, alpha coherences in fronto-posterior (control: 0.30 ± 0.02, aMCI: 0.24 ± 0.01; *p* < 0.01), right temporo-posterior (aMCI: 0.29 ± 0.01, control: 0.36 ± 0.01; *p* < 0.01) were significantly lower for subjects with aMCI than that with normal cognitive function (Figure 4A). No significant differences in inter-regions coherence were found between both groups. The significant different inter-region coherence obtained was then correlated with those neuropsychological measures in which there were differences between controls and aMCI subjects. The alpha coherences in fronto-posterior (*r* = 0.496, *p* = 0.009) and right temporal-posterior (*r* = 0.691, *p* = 0.0002) regions were positively correlated to the scores of MoCA (see Figure 4B). There was no significant correlation between alpha coherences and other neuropsychological tests (data not shown).

**Figure 5:**
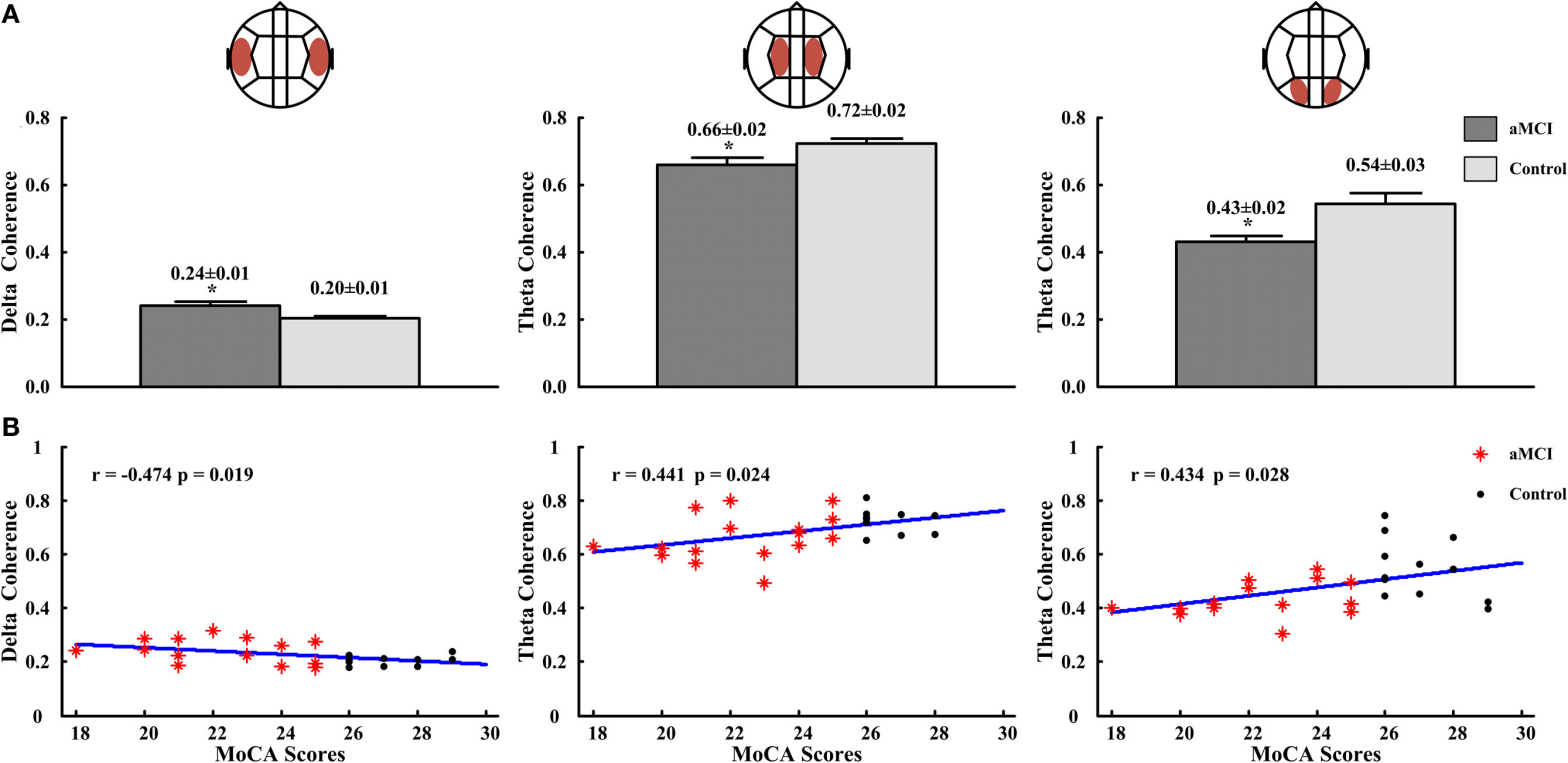
**The delta coherences in LT-RT region and theta coherences in LC-RC and LP-RP regions, and the correlations for the delta coherences or (and) theta coherences with the scores of MoCA. (A)** The delta coherences in LT-RT region (left panel) and the theta coherences in LC-RC (middle panel) and LP-RP (right panel) regions. **(B)** The correlation between the delta coherences and the scores of MoCA in LT-RT region (left panel), and the correlations between the theta coherences and the scores of MoCA in LC-RC (middle panel) and LP-RP (right panel) regions. ^*^p < 0.05.

In inter-hemispheric coherence, aMCI patients showed higher coherence values in delta between LT and RT regions (aMCI: 0.24 ± 0.01, control: 0.20 ± 0.01; *p* < 0.05), and a lower coherence in the theta band in left central-right central (LC-RC) areas (aMCI: 0.66 ± 0.02, control: 0.72 ± 0.02; *p* < 0.05)and in left posterior-right posterior (LP-RP) regions (aMCI: 0.43 ± 0.02, control: 0.54 ± 0.03; *p* < 0.05), than the controls (see Figure 5A). There were no significant differences in other inter-hemispheric regions and frequency bands in both groups. The correlation's results between the significant inter-hemispheric coherence and neuropsychological tests which were different in aMCI and controls showed that the delta coherences of left temporal-right temporal (LT-RT) region were negatively correlated to the MoCA scores (*r* = −0.474, *p* = 0.019), and the theta coherences of LC-RC region (*r* = 0.441, *p* = 0.024) and LP-RP region (*r* = 0.434, *p* = 0.028) were positively correlated to the MoCA scores (see Figure 5B).

## Discussion

Type 2 diabetes or impairment of glucose metabolism may increase the risk of cognitive impairment and accelerate the progress from MCI to dementia, and it is up to 80% of patients with AD (Ganguli et al., 2004; Busse et al., 2006; Yaffe et al., 2006; Hussain, 2007; Xu et al., 2010; Tuma, 2012; Roberts et al., 2014).

The present study examined whether the ratios of power and the values of coherence of rsEEG data can be used to distinguish aMCI from controls with diabetes. Our findings showed that the ratios of power in theta band vs. power in alpha band [P(theta)/P(alpha)] in the frontal region and left temporal region were significantly higher in aMCI subjects. Besides this, the aMCI group showed lower values of coherence in the alpha band in posterior, fronto-right temporal/fronto-posterior/right temporo-posterior regions and in the theta band in LC-RC and LP-RP regions than the control group. Finally, the aMCI patients exhibited an increase in coherence in delta band in LT-RT regions, compared to the control subjects.

In previous studies, rsEEG absolute power were directly used to evaluate cognitive status of MCI subjects (Luckhaus et al., 2008), and specially could reflect neurodegenerative processes in aMCI (Huang et al., 2000; Jelic et al., 2000; Koenig et al., 2005; Babiloni et al., 2006). There were decreased alpha (8–10.5 Hz) power in the parieto-occipital regions (Jelic et al., 1996; Babiloni et al., 2000, 2006; Jelic et al., 2000), significantly increased theta power in the frontal and temporo-parietal regions (Johnson, 2006) and increased delta power widespread over the brain for MCI patients (Babiloni et al., 2006). And the significant correlations between power and neuropsychological assessment scores indicated that aMCI was associated with disruptions in the operation of neuro-cognitive networks (Cummins et al., 2008). Brismar (2007) suggested that the theta rhythm increased in frontal and left central regions, and alpha, beta, and gamma power decreased in temporal region. And it was found that the alpha and beta power was lower in MCI (Rodriguez et al., 2011). The increased slow rhythm power and the reduced fast rhythm power in type 2 diabetic may be associated with cortical damage. However, in this study the absolute power of rsEEG at the different frequency bands was not significantly different between the diabetic aMCI and controls (not shown in the Results section). The possible reason is the rsEEG absolute power was sensitive to non-diabetic's brain performances. The relative power and coherence of rsEEG could be better to indicate aMCI in diabetes.

The relative power of theta and alpha bands was reported as an important predictor for MCI, which correctly classified MCI subjects of 85% (Jelic et al., 2000). However, in this study the relative power for each frequency band was not significantly different between the diabetic aMCI and control groups. Because of the complex structure and richest connections with the hippocampus (Johnson, 2006; Moretti et al., 2007b), the frontal and temporal regions were more sensitive than other regions. Moreover, it has been reported that significant increase in the theta/alpha1 ratio was indicative of cerebrovascular damage (CVD) (Moretti et al., 2007a,b). Our subjects were all diabetic and may be affected by CVD. This may be the reason to support the difference between this study and previous studies. The effects of diabetes on degenerative and CVD may accelerate onset of MCI (Roberts et al., 2014), as insulin-related effects may affect cognitive function (Craft, 2007). Insulin resistance and hyperinsulinemia increased brain intra-neuronal β-amyloid deposition and hyperphosphorylation of tau (Craft, 2005). And the dysregulation of brain insulin signaling may lead to impaired central glucose homeostasis and neurodegeneration. Vascular damage of the brain resulted from diabetes (Craft, 2005; Debette et al., 2011) may contribute to the risk of aMCI (Arvanitakis et al., 2006; Knopman and Roberts, 2010; Roberts et al., 2011). It has been demonstrated that vascular lesions interrupt the cortical cholinergic pathways which may lead to depletion of acetylcholine, resulting in cognitive impairment (Mitrushina et al., 1999). Therefore, this study suggested that the ratios of power at some brain areas can be used as a sensitive index to distinguish aMCI from subjects with normal cognitive function.

Coherence can reflect functional interactions between neural networks (Hogan et al., 2003). In Gomez et al. (2009), it was reported that coherence was lower in all frequency bands in MCI group, and has been used to detect the brain dysfunction thus discriminating MCI patients from controls. In this study, we found that: the alpha coherence decreased in posterior region, fronto-posterior and right temporo-posterior regions; the theta coherence decreased in left and right central and left and right posterior region; and the delta coherence increased in left and right temporal regions in aMCI subjects compared with controls. There are some differences between these findings and the reports in Moretti et al. (2008). The decreased alpha coherence in fronto-posterior and temporo-posterior regions and increased delta in left and right temporal regions have been also reported in previous MCI's studies (Jelic et al., 1996; Jeong, 2004; Babiloni et al., 2008; Moretti et al., 2008). The coherence changes in the alpha and delta bands were associated with aMCI and CVD (Moretti et al., 2008), and authors suggested that the increase of inter-hemispheric coherence in the temporal region was linked to hippocampal atrophy, whereas the decrease of coherence in fronto-parietal regions was linked to subcortical CVD (Moretti et al., 2008). But for other frequency bands, there was regional difference or has no difference between our and their studies (Jeong, 2004; Moretti et al., 2008). In conclusion, this study suggested that the decreased theta, alpha coherence and increased delta coherence in corresponding regions may distinguish aMCI from controls in diabetic.

The results of correlations between scores of MoCA and rsEEG biomarkers indicated that MoCA scores and rsEEG biomarkers among frontal, temporal, and posterior regions are well-correlated. The correlations between other significantly different neuropsychological items and rsEEG biomarkers were not significant. It is worth mentioning that the correlation between the scores of MoCA and alpha coherence was not significant neither. These results suggested that intra-posterior functional connections in alpha band may be relatively preserved in diabetic aMCI. These correlations confirmed the feasibility and value of our studies aimed at detecting aMCI in diabetes by rsEEG biomarkers. And our results evidenced that the sensitivity of MoCA and its utility in diabetic population were better than MMSE (Alagiakrishnan et al., 2013) since this test was usually correlated with the EEG power/coherence.

This study shows the rsEEG may provide efficient methods to monitor the cortical dysfunction associated with the cognitive decline of diabetic patients. The rsEEG measures may be eventually assumed a role in early detecting aMCI or in guiding diagnosis of aMCI in diabetes. Thus, early intervention can be carried out to slow the development pace of aMCI to AD.

However, these results may still be limited, larger prospective studies are necessary to verify the findings in this study.

## Author contributions

Zhijie Bian: Acquisition, analysis, interpretation of data for the study and drafting the manuscript. Qiuli Li: Acquisition. Lei Wang: Design of the study and acquisition. Chengbiao Lu: Revising it critically for important intellectual content. Shimin Yin: Design of the study, final approval of the version to be published and agreement to be accountable for all aspects of the study in ensuring that questions related to the accuracy or integrity of any part of the study are appropriately investigated and resolved. Xiaoli Li: Design of the study, revising it critically for important intellectual content, final approval of the version to be published and agreement to be accountable for all aspects of the study in ensuring that questions related to the accuracy or integrity of any part of the study are appropriately investigated and resolved.

### Conflict of interest statement

The authors declare that the research was conducted in the absence of any commercial or financial relationships that could be construed as a potential conflict of interest.
